# Effect of Maternal Obesity in Mice on IL-6 Levels and Placental Endothelial Cell Homeostasis

**DOI:** 10.3390/nu12020296

**Published:** 2020-01-22

**Authors:** Tobias Kretschmer, Merle Schulze-Edinghausen, Eva-Maria Turnwald, Ruth Janoschek, Inga Bae-Gartz, Peter Zentis, Marion Handwerk, Maria Wohlfarth, Astrid Schauss, Eva Hucklenbruch-Rother, Jörg Dötsch, Sarah Appel

**Affiliations:** 1 Department of Pediatrics and Adolescent Medicine, Faculty of Medicine and University Hospital Cologne, University of Cologne, Kerpener Strasse 62, 50937 Cologne, Germany; merle.schulze.edinghausen@googlemail.com (M.S.-E.); eva-maria.turnwald@uk-koeln.de (E.-M.T.); ruth.janoschek@uk-koeln.de (R.J.); inga.bae-gartz@uk-koeln.de (I.B.-G.); marion.handwerk@uk-koeln.de (M.H.); maria.wohlfarth@uk-koeln.de (M.W.); eva.hucklenbruch-rother@uk-koeln.de (E.H.-R.); joerg.doetsch@uk-koeln.de (J.D.); sarah.appel@uk-koeln.de (S.A.); 2 Center for Molecular Medicine Cologne (CMMC), University of Cologne, Robert-Koch-Strasse 21, 50931 Cologne, Germany; 3 Cologne Excellence Cluster on Cellular Stress Responses in Aging-Associated Diseases (CECAD), Core Facility Imaging, University of Cologne, Joseph-Stelzmann-Strasse 26, 50931 Cologne, Germany; peter.zentis@uni-koeln.de (P.Z.); aschauss@uni-koeln.de (A.S.)

**Keywords:** pregnancy, inflammation, placental vascular insufficiency, placental dysfunction, epigonadal white adipose tissue, human placental vein endothelial cells, IL-6-induced senescence, pregnancy outcomes

## Abstract

Obesity during pregnancy is a known health risk for mother and child. Since obesity is associated with increased inflammatory markers, our objectives were to determine interleukin-6 (IL-6) levels in obese mice and to examine the effect of IL-6 on placental endothelial cells. Placentas, blood, and adipose tissue of C57BL/6N mice, kept on high fat diet before and during pregnancy, were harvested at E15.5. Serum IL-6 levels were determined and endothelial cell markers and IL-6 expression were measured by qRT-PCR and western blot. Immunostaining was used to determine surface and length densities of fetal capillary profiles and placental endothelial cell homeostasis. Human placental vein endothelial cells were cultured and subjected to proliferation, apoptosis, senescence, and tube formation assays after stimulation with hyperIL-6. Placental endothelial cell markers were downregulated and the percentage of senescent endothelial cells was higher in the placental exchange zone of obese dams and placental vascularization was strongly reduced. Additionally, maternal IL-6 serum levels and IL-6 protein levels in adipose tissue were increased. Stimulation with hyperIL-6 provoked a dose dependent increase of senescence in cultured endothelial cells without any effects on proliferation or apoptosis. Diet-induced maternal obesity led to an IUGR phenotype accompanied by increased maternal IL-6 serum levels. In the placenta of obese dams, this may result in a disturbed endothelial cell homeostasis and impaired fetal vasculature. Cell culture experiments confirmed that IL-6 is capable of inducing endothelial cell senescence.

## 1. Introduction

Maternal obesity during pregnancy is a major risk factor for the health of mother and child. Multiple cohort studies exist, demonstrating that the incidence of developing pregnancy associated diseases, e.g., gestational diabetes, preeclampsia, and thromboembolytic complications is increased [1], while the fetus is more endangered of being affected by, e.g., growth abnormalities, such as large for gestational age and small for gestational age (LGA and SGA) [2,3] and stillbirth [4]. Moreover, children exposed to maternal obesity during pregnancy are more likely to develop obesity, diabetes, and other related diseases in later life [5]. Many studies are trying to explain how and why maternal obesity leads to the pregnancy complications described above, often speculating about a dysfunctional placenta. The placenta is the central organ during pregnancy, connecting mother and fetus, providing the fetus with nutrients, oxygen, various hormones, and other important factors. Disturbed placental development and function leads to an imbalance in fetal supply, thereby most likely causing the known fetal complications connected to maternal obesity.

Vascularization of the placenta plays an important role in the capacity of the organ to deliver optimal fetal supply. Several pregnancy complications have been connected to changes in the placental vascular status, such as intrauterine growth restriction (IUGR) and preeclampsia. Moreover, maternal obesity has been described to exert negative effects on the placental vasculature, both in humans [6,7] and various animal species ([8] (mouse); [9] (sheep); [10] (rat); [11] (rat)). As a potential underlying mechanism, low grade systemic inflammation persisting in obese women is discussed, originating from both the adipose tissue and the placenta [12]. Longitudinal studies suggest that, amongst others, IL-6 is increased in pregnant women with higher BMI especially in the second and third trimester [13,14,15]. IL-6 can have both pro- and anti-inflammatory properties depending on the receptor signaling. Classic IL-6 receptor (IL-6R)-mediated signaling is considered to have anti-inflammatory effects while trans-signaling via a soluble IL-6R and gp130 has pro-inflammatory activities [16]. Indeed, it has been shown that pro-inflammatory IL-6 signaling can induce apoptosis in endothelial cells (EC) in vitro [17,18], representing a potential cause for EC damage in vivo and impaired vascularization of the placenta in maternal obesity.

In this project, we aimed to investigate the effect of maternal obesity induced by a high fat diet (HFD) on placental vascularization, EC homeostasis, and proposed that IL-6 causes EC damage leading to placental insufficiency.

## 2. Materials and Methods 

### 2.1. Animal Procedures and Tissue Preparation

All procedures in this study were performed according to protocols approved by the German regulations and legal requirements and were also approved by the local government authorities (Bezirksregierung Köln; LANUV NRW, Germany). The approval and project identification code is 84-02.04.2014.A057/84-02.04.2016.A046. Shortly, C57BL/6N mice (Janvier Labs, Le Genest-Saint-Isle, France) were bred at the animal facility of the Department of Pharmacology of the University of Cologne. Mice were randomly assigned to receive either a control diet (SD, R/M-H, crude fat 33 g/kg, ssniff^®^, Soest, Germany) or a fat rich diet (HFD, C1057 modified, crude fat 351 g/kg Altromin, Lage, Germany) ad libitum which contains fatty acids and calories from lipids in excess (Supplementary Materials, Table S1). For detailed information on animal housing and experimental settings see [19,20]. 

Female mice were kept on the respective diet after weaning at the age of three weeks until the end of experiments. Mating started at the age of 12 weeks if females of the SD group were lighter than 23 g and HFD group females were heavier than 23.5 g, signifying an obese phenotype as reported previously [19,21]. Pregnant dams used for stereological analyses were injected i.p. with 10 µL/g body weight of a 10 mg/mL BrdU-solution (Sigma, Steinheim, Germany, dissolved in phosphate-buffered saline, PBS, Dulbecco w/o calcium and magnesium) 1.5 h before sacrifice, and received an additional 0.1 mg/kg bodyweight buprenorphine (Bayer, Vital GmbH, Leverkusen, Germany) dissolved in 0.9% (w/v) sodium-chloride solution (Fresenius Kabi Deutschland GmbH, Bad Homburg, Germany) 30 min before sacrifice due to German regulations and legal requirements. After sacrifice, blood was taken via cardiac puncture in order to collect serum and organs were harvested for further examination. Briefly, a caesarean section was performed and the uterus was removed. Each feto-placental unit was opened, the fetus was removed, weighed, and decapitated and the placenta was dissected for either RNA/protein isolation (cord, amnion sac, and connective tissue were carefully detached) or histological examinations (the uterus part attached to the placenta was not removed in order to not destroy the decidua). Then, epigonadal white adipose tissue (egWAT) was dissected. Both placenta samples for RNA/protein isolation and egWAT samples were immediately quick frozen in liquid nitrogen and stored at −80 °C until further processing. Whole placentas (for stereological analysis) or placental halves (for immunofluorescence and immunohistochemistry stains), were fixed for 24 h in 4% phosphate-buffered formalin (Roti^®^-Histofix, Carl Roth, Karlsruhe, Germany), incubated for 24 h in 70% isopropanol and were subsequently embedded in paraffin.

### 2.2. qPCR Analysis

qPCR analysis was performed as described before [22,23]. Briefly, total RNA was isolated from the placenta or egWAT samples with a TriReagent^®^ solution (Sigma, Steinheim, Germany) and 1 µg was converted into cDNA. 2.5 µL of cDNA was used in a TaqMan assay (Platinum^®^ qPCR SuperMix-UDG with Rox, Invitrogen, Carlsbad, CA, USA) to detect expression levels of IL-6, CD31 and vWF in the placenta, or IL-6 in egWAT. For Tie-1, we used 1 µL of cDNA in a SYBR assay (GoTaq qPCR Master Mix, Promega, Madison, WI, USA). For normalization, HPRT or β-actin expression levels were assessed. Oligonucleotides used for the analyses are listed in Supplementary Materials, Table S2. Results were analyzed using the deltadeltaCT method, expressed as fold-induction compared to the corresponding control group. Analysis was performed on multiple placentas per dam as indicated in the figure legend.

### 2.3. Protein Isolation and Western Blot Analysis

Tissue samples were processed as described previously [22] to perform western blot analyses. Primary and secondary antibodies used to detect specific targets on the blotting membrane were diluted as indicated in Supplementary Materials, Table S3. Specific bands were detected on the membranes with the BIORAD molecular imager ChemiDOC TM XRS + imaging system (Bio-Rad Laboratories, Munich, Germany) and densitometrically quantified using the Image Lab software version 5.2.1 (Bio-Rad Laboratories). Details on the method can be found in [22]. Analysis was performed on multiple placentas per dam as indicated in the figure legend.

### 2.4. Histological Stainings (Immunohistochemistry, Immunofluorescence, Stereology)

For immunohistochemistry (IHC), sections were taken from paraffin-embedded placental halves close to the placental midline and the umbilical cord at 5 µm thickness. Immunofluorescence (IF) stains were performed on 3 µm thick sections from the same placental region. Stereological samples were randomly oriented before exhaustively sectioning each sample on a rotary microtome (Leica RM2) at 7 µm thickness. A random section within the first 40 sections was chosen as a start. From there, three consecutive sections were collected, and every 40 sections afterwards another three sections were collected until the sample was exhausted. Sections were collected on SuperFrost slides (Thermo Scientific, Braunschweig, Germany), deparaffinized in NeoClear^®^ (Merck, Darmstadt, Germany), rehydrated using increasing concentrations of ethanol (Carl Roth, Karlsruhe, Germany) and rinsed in distilled water. Antigen unmasking was performed in citrate buffer pH 6 (DAKO, Glostrup, Denmark) for 25 min using a steam heater (FS 20, Braun, Germany), followed by an endogeneous peroxidase block. IF stain sections were further incubated with 300 mM glycine (Carl Roth, Karlsruhe, Germany) in PBS for 30 min. Sections were subsequently blocked with Sea Blocking buffer (Thermo Fisher, Rockford, IL, USA) before primary antibodies (Table S3) were incubated overnight at 4 °C. For IF stains, fluorochrome-labelled secondary antibodies (Table S3) were applied the next day for 1 h. Sections for CD31—phosphoS139-gammaH2A.X co-IF stains, which were first stained for CD31, were re-stained for phosphoS139-gammaH2A.X according to the instructions of the Tyramide SuperBoost™ kits with Alexa Fluor™ (Invitrogen, Eugene, OR, USA). Briefly, sections were unmasked, peroxidase-blocked, incubated with glycine, sea-blocked, and incubated with primary and secondary antibodies as mentioned above. Afterwards, nuclei were stained with DAPI and sections were mounted with an aqueous mounting medium (DAKO, Glostrup, Denmark). For stereology or IHC, sections were incubated with a HRP-coupled secondary antibody, before incubation with a DAB solution (ImmPACT™ DAB Substrate Kit, SK-4105, Vector Laboratories, Burlingame, CA, USA) or AEC (permanent AEC kit, Zytomed Systems, Berlin, Germany), respectively. Subsequently, a counterstain with Mayer’s hematoxylin (Carl Roth, Karlsruhe, Germany) was performed. Slides were washed in tap water, dehydrated in alcohol, NeoClear^®^, and mounted in NeoMount^®^ (Merck, Darmstadt, Germany). Stained sections were recorded using either a slide scanner (Leica SCN400, for IHC and stereology) or the EVOS FL Auto2 or Leica TCS SP8 microscopes (for IF). Images for figures were processed with Adobe Photoshop CS2. Using a stereological approach, volumes, surface areas, and lengths of complex tissues were determined from histological sections [24].

### 2.5. Analysis of IF Sections

For the analysis of IF sections, 6–7 scans of placentas per group were used, and each of four independent investigators was blinded for the complete procedure. Each scan of a whole placental section was divided into 10 equally sized frames and three frames, i.e., one frame close to the middle, one close to the outside end, and one in between middle and outside end, were counted. BrdU, gammaH2AX, and TUNEL positive and negative EC nuclei were manually counted using the cell counter tool in ImageJ Fiji [25]. Total cell number was automatically counted in the DAPI channel using particle analysis in ImageJ Fiji. Four independent investigators performed the analysis and provided similar results. Therefore, representative results are shown from one of the four investigators.

### 2.6. Stereological Analysis of Sections

For the analysis of labyrinth zone (lz) structures, each of four independent investigators was blinded for the complete procedure. A lz image was extracted from whole scans of placental sections according to manually drawn outlines. The lz image was fractioned into 4096 × 4096 pixel tiles (3.96 µm/pixel). Maternal blood sinus (MBS), fetal capillary (FC), and trophoblast volume densities were obtained by point-line grids of a defined area per point and line length. The number of points falling on MBS, FC, and trophoblast, and the amount of intersections of the lines with a MBS and FC were counted on two random tiles per lz image. From these values the volume densities (V_V_) were calculated as mentioned above and multiplied by total lz volume to obtain absolute quantities. Surface area was calculated using the following equation:

S_(structure)_ = (2 × Σ I_(structure)_/I_(p)_ × Σ P_(reference)_) × V_(reference)_ (1)

Σ I_(structure)_ represents the sum of intersections of the line with the structure (MBS or FC), Σ P_(reference)_ is the total number of points on the lz, and I_(p)_ is the length of the line associated with each grid point [26]. These surface area densities were then multiplied by the reference volume of the lz, which is derived from the manually outlined scans in µm^2^, and the total number of sections that showed lz plus an extra 40 sections for correction, multiplied by 7 µm section thickness, to calculate absolute surface areas. To determine capillary length densities, a counting frame with two contiguous forbidden lines [27] was superimposed on the lz of whole scans. The total number of fetal capillaries within the frame, that did not touch the forbidden lines, were counted and the total number of frames on the lz was recorded. Via calculation of numerical density of capillary profiles per unit area of lz, length density of FC, and multiplying by the reference volume of the lz as described above, absolute capillary length and capillary diameter were derived [28,29]. 

Stereology data were collected in Microsoft Excel and data from each placenta were weight-averaged. The cell counter tool of ImageJ Fiji was used to count grid points and line intersections in each picture. Four independent investigators performed the analysis and provided similar results. Therefore, representative results are shown from one of the four investigators.

### 2.7. IL-6 ELISA

IL-6 levels were measured in undiluted maternal serum samples with the mouse IL-6 ELISA kit (EZMIL6) from Merck Millipore (Darmstadt, Germany) following manufacturers’ protocol. 

### 2.8. Cell Culture Assays

Human placental vascular endothelial cells (HPVECs) were purchased from ScienCell (#7100, Carlsbad, CA, USA) and cultured in an endothelial cell medium (ScienCell, #1001) supplemented with 1% endothelial cell growth supplements (ScienCell, #1052), 5% fetal bovine serum (ScienCell, #0025), and 1% penicillin/streptomycin (ScienCell, #0503). For detecting apoptosis, 15,000 cells/well were seeded in 96-well plates coated with gelatin and cultured for 12 h. Then, stimulation of cells with 10 or 50 ng/mL hyperIL-6 (recombinant human IL-6/IL-6R Chimera, #8954-SR, R&D Systems, Minneapolis, MN, USA) was performed for 12 h before measuring Caspase-3/7 activity with the Caspase-Glo^®^ 3/7 Assay Systems assay (# G8090, Promega, Madison, WI, USA) following the manufacturers’ protocol. To measure proliferation, cells were seeded in 96-well plates coated with gelatin at a density of 10,000 cells/well and cultured for 12 h. Then, cells were stimulated with or without hyperIL-6 for 12 h, before adding fresh stimulation medium containing BrdU for additional 24 h. Detection of incorporated BrdU was measured following the manufacturers’ protocol (#11 647 229 001, Roche, Mannheim, Germany). For assessing cell senescence, we seeded 10,000 cells/well in a gelatin-coated 96-well plate and incubated the cells for 12 h. Then, hyper-IL-6 stimulation was performed for 48 h. Senescence-associated beta-galactosidase activity was detected by a protocol published by [30]. Finally, tube formation capacity was assayed by a classical tube formation assay. The wells of an ibidi angiogenesis µ-slide (#81506, ibidi, Graefelfing, Germany) were coated with 10 µL growth factor reduced matrigel (#35 62 30, BD Biosciences, Bedford, MA, USA) before plating 5000 cells/well in stimulation medium. Cells were then incubated for 12 h under a confocal laser scanning microscope (Zeiss Meta 510) equipped with an incubation chamber to keep the cells at 37 °C and 5% CO_2_. After 12 h, formed tubes were imaged and analyzed with ImageJ Fiji. The number of closed tubes were counted and normalized to the total area analyzed. 

### 2.9. Statistical Analysis

Statistical analysis was performed with the GraphPad Prism 7 Software. All values in the text are mean ± SEM. Normality distribution was tested using the D’Agostino and Pearson test. Differences were evaluated using a two-tailed Student’s t-test (for normal distributed data) or a Mann–Whitney test (for non-normal distributed data). Significant differences were taken at the *p* < 0.05 level.

## 3. Results

Previously, we reported that fetal weight was significantly reduced in HFD-induced maternal obesity (0.3786 ± 0.004382 g vs. 0.4482 ± 0.008337 g, *p* < 0.0001) at G15.5 [19]. In this project, we therefore aimed to determine whether this IUGR phenotype is caused by a dysfunctional vascularization of the placenta due to maternal obesity. 

### 3.1. Downregulation of Endothelial Cell Markers in Placentas of Obese Dams

**Figure 1:**
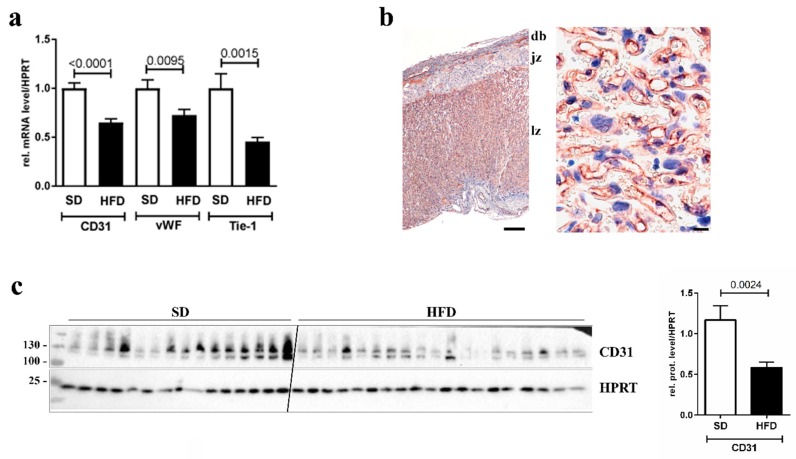
Effect of high fat diet (HFD) on placental endothelial cell markers. (**a**) qPCR analysis of endothelial cell markers CD31, von Willebrandt factor (vWF), and Tie-1, normalized to hypoxanthine-guanine phosphoribosyltransferase (HPRT), in whole placenta lysates of control (SD) and obese (HFD) mice. The *p*-value is indicated in the graph. *n* = 21 placentas from five dams for SD and *n* = 21 placentas from six dams for HFD. (**b**) A representative immunohistochemical staining of CD31 in a control (SD) placenta. Left image: Overview of a section through a whole placenta; note that CD31 is mainly located in the labyrinth zone (lz), not in the junctional zone (jz) or the decidua basalis (db). Scale bar 200 µm. Right image: Magnification of the lz; note that CD31 is located in fetal capillaries. Scale bar 10 µm. (**c**) Western blot analysis of CD31 in whole placenta lysates of SD and HFD mice. HPRT was detected for normalization. Both bands in the images were quantified by densitometry and the relative amount of CD31/HPRT in the two groups is indicated in the bar graph next to the western blot. The *p*-value is indicated in the graph above the bars. *n* = 15 placentas from five dams for SD and *n* = 19 placentas from seven dams for HFD.

To examine whether vascularization of the placenta could be affected by maternal obesity, we first studied mRNA expression and protein levels of EC markers in total placenta lysates from G15.5 mice. In a qPCR assay, the endothelial markers cluster of differentiation 31 (CD31, also known as PECAM-1 (platelet endothelial cell adhesion molecule-1)), von Willebrandt factor (vWF), and Tyrosine kinase with immunoglobulin-like and EGF-like domains-1 (Tie-1, an Angiopoietin receptor) were measured and normalized to HPRT (similar results were obtained for other control genes, data not shown). All EC markers were significantly downregulated in placentas from obese dams (Figure 1a). CD31 is mainly expressed in the labyrinth zone of the murine placenta where nutrient and gas exchange takes place (Figure 1b). CD31 was also significantly reduced on the protein level in placentas from HFD fed animals (Figure 1c). Hence, our data suggest that ECs of the placental transfer zone might be affected by maternal obesity.

### 3.2. Endothelial Cell Homeostasis and Vessel Structure

**Figure 2:**
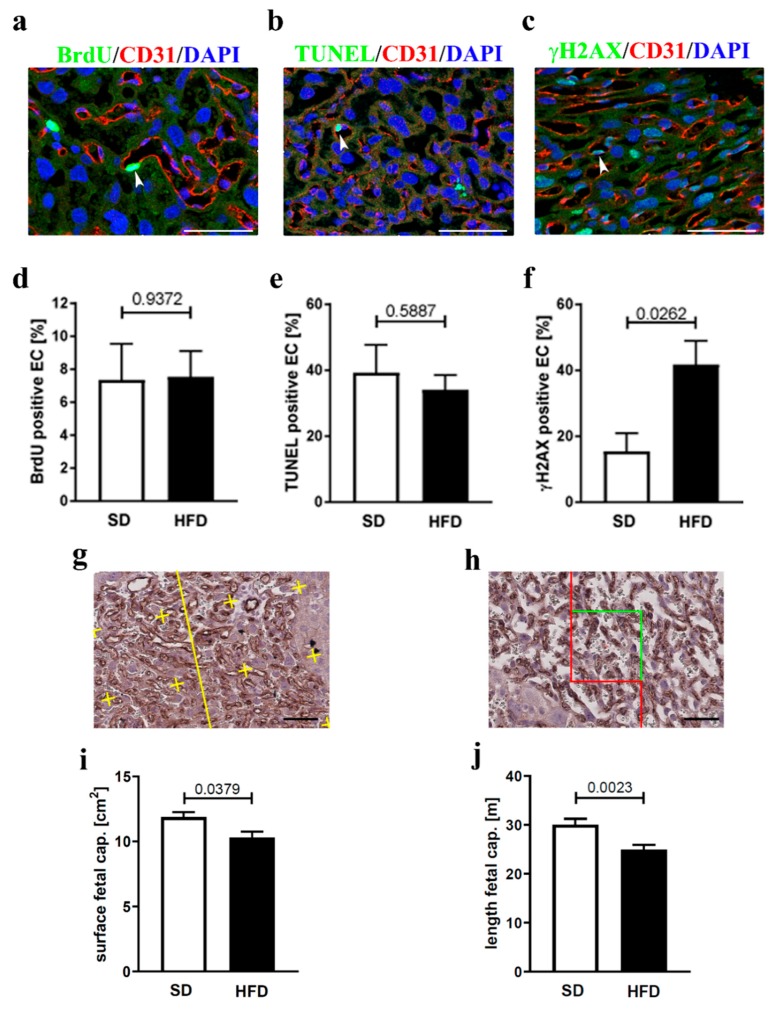
Effect of HFD on placental endothelial cell homeostasis and vessel structure. (**a**) Representative image of an IF co-staining of the placental lz from controls (SD) with BrdU (green), CD31 (red), and 4′,6-diamidino-2-phenylindole (DAPI) (blue). A BrdU-positive CD31-positive cell is marked with an arrow. Scale bar 50 µm. (**b**) Same as (**a**), but TUNEL (green), CD31 (red), and DAPI (blue) co-staining. (**c**) Same as (**a**), but with gammaH2AX (green), CD31 (red), and DAPI (blue) co-staining. (**d**–**f**) Quantification of either BrdU (*n* = 6 placentas, one placenta per dam, for both SD and HFD), TUNEL, or gammaH2AX (*n* = 7 placentas, one per dam, for both SD and HFD) positive endothelial cells (EC) in the lz of placentas from either control (SD) or obese (HFD) dams. The *p*-value is indicated in the graph. (**g**) Representative image of the lz of a control (SD) placenta, stained with an antibody against CD31. Lines and crosses (yellow) are necessary to assess the surface of fetal capillaries (CD31-positive vessels). Scale bar 50 µm. (**h**) Same as (**g**), but with rectangles (green and red lines) to assess length of fetal capillaries. (**i**) Quantitative analysis of fetal capillary surface [cm^2^] in the placental lz of SD (*n* = 8 placentas from eight dams) and HFD (*n* = 7 placentas from seven dams) mice. The *p*-value is indicated in the graph. (**j**) Quantitative analysis of fetal capillary length [m] in the placental lz of SD (*n* = 8 placentas from eight dams) and HFD (*n* = 7 placentas from seven dams) mice. The *p*-value is indicated in the graph.

We next assessed EC homeostasis in the labyrinth zone of placenta sections (midline) by performing immunohistochemical co-stainings of CD31 (to detect ECs) and either BrdU (proliferation marker), TUNEL-staining (apoptosis marker), or gammaH2AX (senescence marker) (Figure 2a–c). Next, the number of either BrdU-, TUNEL-, or gammaH2AX positive ECs was quantified. While there was no difference in BrdU- or TUNEL-positive ECs in the labyrinth zone between the two test groups (Figure 2d,e), we detected significantly more gammaH2AX-positive ECs in the labyrinth zone of obese dams (Figure 2f), indicating a higher senescence rate of this cell type in maternal obesity. To understand if vessel structure of the labyrinth zone was altered in HFD fed dams, we performed stereological analyses of the labyrinth zone of CD31-stained histological placental sections. Our data clearly showed that fetal vessel surface and fetal capillary length was significantly decreased in the labyrinth zone of placentas from HFD animals (Figure 2g–j).

### 3.3. IL-6 Inflammation Marker Levels

**Figure 3:**
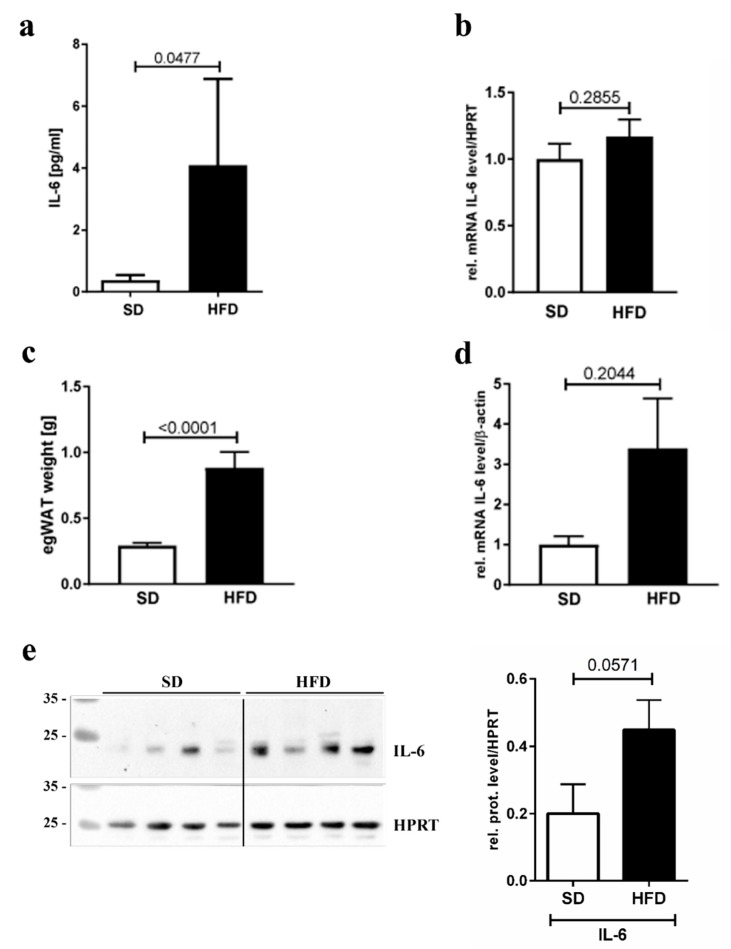
Effect of HFD on IL-6 level in blood, placenta, and egWAT. (**a**) IL-6 circulating levels in maternal serum, measured by an ELISA, in SD (*n* = 9) and HFD (*n* = 10) dams. (**b**) IL-6 mRNA expression level, normalized to HPRT and measured in whole placenta lysates by qPCR. *n* = 16 placentas from four dams for SD and *n* = 25 placentas from seven dams for HFD. (**c**) Fat pad weight of epigonadal white adipose tissue (egWAT). *n* = 12 for SD and 14 for HFD. (**d**) qPCR analysis of IL-6 mRNA levels, normalized to beta-actin, in egWAT. *n* = 8 for SD and HFD. (**e**) Western blot analysis of IL-6 levels in egWAT lysates. HPRT was detected for normalization. Relative IL-6/HPRT levels, quantified by densitometry, are indicated in the bar graph next to the western blot. The *p*-value is indicated in the graph above the bars. *n* = 4 for SD and HFD.

To test whether IL-6 was upregulated in maternal obesity, we first quantified circulating amounts of IL-6 in maternal serum and detected a significant four-fold increase in IL-6 serum levels in HFD-fed dams (Figure 3a). As shown by qPCR analysis, IL-6 mRNA expression in the placenta was not increased under HFD feeding (Figure 3b), excluding this organ as origin of increased circulating IL-6 levels. As an alternative IL-6 source, we assessed egWAT, the fat pad surrounding the uterus. This fat pad was significantly heavier in obese compared to lean dams (Figure 3c). Indeed, in egWAT, IL-6 mRNA levels were increased about 3-fold. However, the difference failed to reach statistical significance (Figure 3d). We also measured IL-6 protein levels in egWAT by western blot, demonstrating about 2.5-fold higher levels in obese dams, again without reaching statistical significance (Figure 3e). Hence, in our mouse model circulating IL-6 levels are increased in obese dams, probably due to higher IL-6 secretion by the egWAT.

### 3.4. IL-6 Stimulation of Placental Endothelial Cells

**Figure 4:**
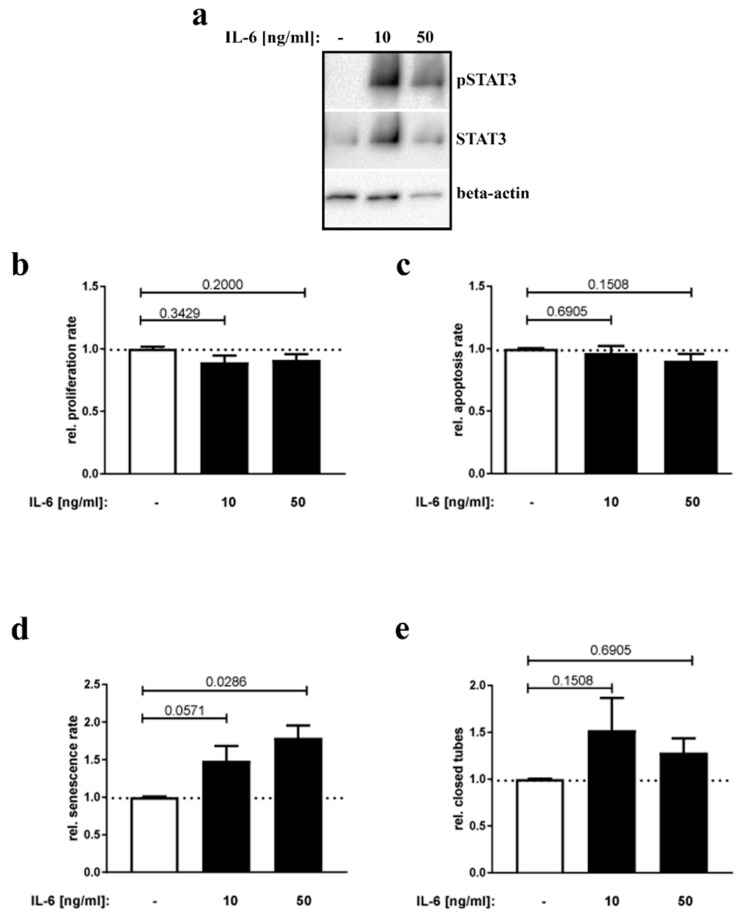
HPVEC homeostasis and function under hyperIL-6 stimulation. (**a**) Western blot analysis of HPVEC cells stimulated with the indicated concentrations of hyperIL-6 for 15 min. Detected were signals of phospho-STAT3, STAT3, and beta-actin with specific antibodies as indicated. (**b**) HPVECs were stimulated with either 10 or 50 ng/mL hyperIL-6 or no hyperIL-6 as control and relative proliferation rate was assessed with a BrdU-assay. *n* = 6 replicates. (**c**) Same as (**a**), but measurement of relative apoptosis rates via a Caspase-3/7 activity assay. *n* = 5 replicates. (**d**) Same as (**a**), but detection of relative senescence rates by determining senescence-associated beta-galactosidase activity. *n* = 5 replicates. (**e**) Analysis of tube formation capacity under the influence of 10 or 50 ng/mL hyperIL-6. *n* = 5 replicates (**b**–**e**) *p*-values are indicated in the graphs.

IL-6 crosses the placental barrier [31,32] and passes fetal ECs in the labyrinth zone. Since IL-6 has been described to cause EC activation and apoptosis [17], we tested in cell culture assays whether hyperIL-6 is capable of altering placental EC homeostasis and/or function. For this purpose, HPVECs (primary endothelial cells isolated from human placenta) were incubated with two different concentrations of hyperIL-6. HyperIL-6 is a bioactive fusion protein consisting of recombinant human IL-6 (Val30-Met212) that is bound by a small glycine and serine rich peptide chain (GGGSGGGSGGGS) to IL-6R-alpha (Leu20-Asp358) [33]. First, activity of hyperIL-6 was verified by increased phospho-STAT3 levels, a well-known target of IL-6 signaling (Figure 4a). Next, we quantified hyperIL-6 effects on proliferation (BrdU), apoptosis (Caspase-3 GLO assay), and senescence (senescence-associated beta-Galactosidase activity). Moreover, tube formation capacity was determined as an indicator of endothelial function. No changes regarding proliferation, apoptosis, or tube formation capacity were detected (Figure 4b,c,e). However, senescence of HPVECs was significantly increased following hyperIL-6 stimulation (Figure 4d). 

## 4. Discussion

Here, we demonstrate that HFD-induced maternal obesity leads to downregulation of EC markers and impaired fetal vasculature in the feto-maternal transfer zone of the placenta. Furthermore, we postulate that EC damage is due to increased senescence rates, possibly induced by elevated IL-6 levels in maternal blood, originating most likely from WAT.

Fetus position within the uterine horn in rodent animal models has been described as an important factor in feto-placental development [34] and the variation in fetal weight per conceptus in our mouse model further supports the rational that each fetus with its respective placenta is biologically distinct. Additionally, it has recently been suggested that dimorphisms, e.g., placental and fetal sex, exist which have an impact on placental development and fetal growth [35,36]. Furthermore, when collecting physiological data from embryos in utero or investigating feto-placental units in genetically modified dams, differences between each feto-placental unit can be observed regarding heart rate [37] or normal fetal development independent of fetal genotype [38], respectively, suggesting that analyzing multiple placentas per dam could be appropriate. Lastly, each placenta has a certain individual reserve capacity to support proper fetal growth that is independent of the dam [39]. Hence, analyzing multiple placentas in qPCR and western blot experiments per dam generates a deeper understanding of individual differences and pinpoints variations per fetus.

The HFD used in this study is sufficient to induce maternal obesity [19], compromised glucose tolerance [40] and significant weight reduction in fetuses at E15.5 (see above). The bulk of its fatty acid content comprises saturated and mono-unsaturated C-16:x and C-18:x fatty acids from palm oil (4.5%) and animal lard (30.5%) which make up a total of about 34% metabolizable energy from fat together with the remaining parts of fatty acids. This composition may not reflect what is generally called a typical western-type diet, which contains higher amounts of saturated fatty acids and sugars [41]. However, maternal and fetal phenotypes resulting from high fat high sugar or HFD show similar features [19,21,42] and HFD animal studies are generally well accepted to study the impact of (maternal) obesity on various organ systems [43].

EC activation and damage has been reported in pre-eclampsia [44] and obesity-induced vascular dysfunction is known to occur in obese patients [45], however to the best of our knowledge there are no studies investigating the impact of maternal obesity on placental EC homeostasis mediated by IL-6 and providing a stereological evaluation of vascular development. This new insight could have clinical implications since proper placental vascular development is necessary to prevent placental dysfunction and hypertension. Earlier studies in humans and mice showed that HFD could have a profound impact on the female reproductive function, and studies in mice and rats suggest distinct consequences of HFD on placental function and pregnancy outcome [46,47,48]. Furthermore, it has been shown that HFD has a significant impact on hemodynamics and vascularization in the placenta [8,49] and by the use of stereological means, it is possible to evaluate the developmental state of the vascular system in the murine placenta [29]. Thus, the observed impaired vascularization of the feto-maternal transfer zone in our model, characterized by decreased expression levels of (fetal) EC markers CD31, vWF, and Tie-1, as well as decreased fetal vessel surface and length, might contribute to fetal growth restriction in obese mothers. From human studies, it is well known that a disturbed placental angiogenesis plays an important role in the development of IUGR [50,51]. Ultimately, our data suggests that dietary lipid intake and composition could have clinical implications for placental vascularization and function and hence, offspring health.

The EC specific receptor tyrosine kinase Tie-1, implicated in angiogenesis and vascular maturation [52], has been reported to be upregulated in inflammatory diseases such as rheumatoid arthritis and atherosclerosis. Furthermore, overexpression of Tie-1 in ECs is capable of inducing an inflammatory response and it might pose a target for anti-angiogenesis treatments in cancer [53,54]. Interestingly, our results showed downregulation of Tie-1 in placental tissue eliminating a potential effect on the observed inflammatory environment mediated through Tie-1. However, since it has been speculated that Tie-1 could be involved in placental vascular modulation and angiogenesis [55], the observed downregulation could further add to the impaired vascular development in our mouse model.

If EC damage in the placenta of our animals is indeed due to increased senescence rates requires further examination, since gammaH2AX (H2AX phosphorylated at serine-139) is not a specific senescence marker, but rather a marker for damaged DNA regions (DSB, DNA double strand breaks [56]) and can be even increased in proliferating cells [57]. A positive gammaH2AX signal in the absence of a positive proliferation or apoptosis signal however, is assumed to indicate senescent cells in tissue samples [58]. Due to technical issues, for which co-staining for CD31 and nuclei was done to count only EC nuclei, we were not able to co-stain for all parameters simultaneously. However, since no changes in proliferation or apoptosis rates of the ECs were detected in the transfer zone of obese dams, we assume that increased gammaH2AX signal is representing DNA damage typical for senescent cells. Interestingly, in a cell culture assay using placental ECs, we were able to show that hyperIL-6 increases senescence rates in a dose-dependent manner in vitro. Indeed, IL-6 is known to induce senescence, e.g., in fibroblasts [59]. Since IL-6 levels are increased in the maternal blood stream of obese dams and it is known to cross the placental barrier [31,32], it is tempting to speculate that IL-6 is responsible for inducing higher senescence rates in fetal capillaries. ECs however do not express the IL-6R [60], but IL-6 has been shown to act also on cells that do not harbor the IL-6R. The gp130 receptor is sufficient to induce signaling, since both IL-6 and the soluble IL-6R circulate in the blood stream and hence induce the so called trans-signaling which is the relevant pro-inflammatory pathway in almost all cell types [16,61].

The precise mechanistic connection between higher circulating IL-6 levels and increased EC senescence in placentas of our model however, remains unclear. Currently, an IL-6 inhibition mouse model of HFD-induced maternal obesity is tested in our lab, in which the soluble IL-6R is targeted for inhibition, to test the hypothesis that EC damage is indeed caused by increased circulating IL-6 levels and gp130 receptor-mediated trans-signaling.

WAT hypertrophy or hyperplasia was apparent from weight gain of epigonadal fat pads in HFD-fed dams and increased levels of IL-6 mRNA and protein were measured in egWAT of those dams. No change in placental IL-6 gene expression was found suggesting that the placenta did not induce autocrine signaling. It is well accepted that WAT produces inflammatory cytokines, such as IL-6, and contributes to an inflamed state in obesity through secretion of such cytokines [62,63]. Thus, it might be possible, that HFD-induced obesity and the resulting significant increase in WAT in our model caused increased IL-6 production and subsequent secretion. Since IL-6 from the serum can ultimately reach placental tissues [31], we speculated that the axis of “WAT—maternal serum—placental labyrinth” represents a possible route of obesity-induced placental inflammation and EC damage leading to placental insufficiency. However, further studies are warranted to decipher the precise mechanisms postulated herein.

## 5. Conclusions

In summary, we believe that this study showed the effect of maternal obesity on placental vascularization and suggested that IL-6-mediated EC senescence could contribute to the observed placental insufficiency thereby adding clinically relevant insight. Presumably, a HFD-induced increase in WAT, which is able to produce and secrete IL-6, could serve as a source for the inflammatory cytokine reaching placental cells. In the light of an increasing prevalence in (maternal) obesity and poor food choice in western societies, we inferred that these results are of nutritional and clinical relevance. Clearly, more investigations need to be conducted to understand further the precise molecular mechanisms of the processes and the influence of various diets and nutrients in this context.

## Supplementary Materials

The following are available online at https://www.mdpi.com/2072-6643/12/2/296/s1, Table S1: Excerpt of nutrient content of the fat rich diet (HFD) used in this study, Table S2: Oligonucleotides used. Listed in the table are the oligonucleotides used in this study, Table S3: Antibodies used. Listed in the table are the antibodies used in this study and the dilution of the antibody used for detecting its antigen in a western blot (WB) or an immunofluorescence (IF) or immunohistochemistry (IHC) assay.
