# Supplementary material for: Effect of Maternal Obesity in Mice on IL-6 Levels and Placental Endothelial Cell Homeostasis
Source: Nutrients. 2020 Jan 22;12(2):296. doi: 10.3390/nu12020296 (PMC7071123; doi:10.3390/nu12020296)
Supplement: Supplementary file 1 [file nutrients-12-00296-s001.pdf]

Table S1. Excerpt of nutrient content of the fat rich diet (HFD) used in this study.

ingredient	unit	content
Crude fat	mg/kg	350,921
Crude protein	mg/kg	204,600
Saccharides	mg/kg	256,291.3
Metabolizable Energy	kcal/kg	5,297.347
Palmitic acid C-16:0	mg/kg	84,800
Palmitoleic acid C-16:1	mg/kg	10,401
Stearic acid C-18:0	mg/kg	48,600
Oleic acid C-18:1	mg/kg	139,135
Linoleic acid C-18:2	mg/kg	31,550
Linolenic acid C-18:3	mg/kg	3,565
Arachidic acid C-20:0	mg/kg	5,200
Eicosaenic acid C-20:1	mg/kg	2,595
Arachidonic acid C-20:4	mg/kg	120

Table S2. Oligonucleotides used. Listed in the table are the oligonucleotides used in this study.

Target gene	forward 5′-3′	reverse 5′-3′	probe 5′-3′
CD31	AAAGCCAAGGCCAAACAGAA	CCAGAAACATCATCATAACCGTAATG	AGATGTCCAGGCCAGCTGCTCCACTT
vWF	GGGAGTTTTTTGACATCCATTTG	TACCCAGCCTCGCGTTCTAG	TGACCAAAGCATCTCCATGCCCTACG
Tie-1	GCTAAAAGAGTATGCGTCTGAAAATG	GGCCCCCAAGAGGTTGATAAT	---
IL-6	ACAAGTCGGAGGCTTAATTACACAT	AATCAGAATTGCCATTGCACAA	TCTTTTCTCATTTCCACGATTTCCCAGAGAA
HPRT	TGGCCATCTGCCTAGTAAAGCT	TAGGCTCATAGTGCAAATCAAAAGTC	TTTTTAGAAATGTCAGTTGCTGCGTCCCC
beta-actin	TGACAGGATGCAGAAGGAGATTACT	GCCACCGATCCACACAGAGT	ATCAAGATCATTGCTCCTCCTGAGCGC

Table S3. Antibodies used. Listed in the table are the antibodies used in this study and the dilution of the antibody used for detecting its antigen in a western blot (WB) or an immunofluorescence (IF) or immunohistochemistry (IHC) assay.

antibody name	company	catalogue number	host species	clonality	dilution (WB)	dilution (IF, IHC)
CD31	Abcam	Ab28364	Rabbit	polyclonal	1:1000	1:300
BrdU	CST	#5292	Mouse	monoclonal	-	1:1,000
gammaH2aX	Abcam	Ab11174	Rabbit	polyclonal	-	1:4,000
IL-6	R&D Systems	MAB406	Rat	monoclonal	1:200	-
phospho-STAT3	CST	#9145	Rabbit	monoclonal	1:1000	-
STAT3	CST	#9139	Mouse	monoclonal	1:2000	-
HPRT	Abcam	Ab10479	Rabbit	polyclonal	1:1,000	-
beta-actin	CST	#3700	Mouse	monoclonal	1:5000	-
anti-mouse-HRP	CST	7076S	Horse	-	1:2,000	-
anti-rabbit-HRP	CST	7074	Goat	-	1:2,000	-
anti-mouse-Al488	Jackson Immuno Research	115-485-003	Goat	-	-	1:400
anti-rabbit-Al488	Jackson Immuno Research	115-485-003	Goat	-	-	1:400
DAPI	Sigma-Aldrich	10236276001	-	-	-	1 µg/mL
Alexa Fluor™ 555 Tyramide SuperBoost™ Kit	Invitrogen	B40923	Goat	-	-	1:500
